# Reverse Migration, the Black Church and Sexual Health: Implications for Building HIV/AIDS Prevention Capacity in the Deep South

**DOI:** 10.3934/publichealth.2016.2.242

**Published:** 2016-04-25

**Authors:** Pamela Payne Foster, Martina Thomas, Dwight Lewis

**Affiliations:** 1. Department of Community and Rural Health, The University of Alabama School of Medicine, Tuscaloosa, Alabama, USA; 2. Anthropology Department, The University of Alabama, Tuscaloosa, Alabama, USA; 3. Institute for Rural Health Research, The University of Alabama, Tuscaloosa, Alabama, USA

**Keywords:** HIV/AIDS, migration, African Americans, Deep South, The Black Church

## Abstract

The Black Church has long been purported as being strongly influential in the lives of Blacks in America. Recent U.S. census data trends highlight a “reverse migration” pattern where Blacks are moving back to the South from larger metropolitan areas in other U.S. geographical regions. This migration pattern parallels the increasing HIV/AIDS prevalence among Blacks in the Deep South. This paper reviews both the historical and current migration patterns among Blacks, as well as the current HIV/AIDS epidemic among Blacks in the Deep South. Thereafter, the authors discuss an existing framework for increasing HIV/AIDS prevention capacity through a conceptual connection of migration, religion and sexual health. The authors use case studies to support the proposed framework. It is hoped that the framework could be used to address HIV/AIDS health disparities and other chronic diseases affecting Blacks in America.

## 1. Introduction

During the 20^th^ century, migration patterns suggest that Blacks were leaving the Deep South to move to other areas in the U.S. Recent migration trends suggest a “reversal” where Blacks are moving back to the Deep South. These more recent migration trends mirror current demographic changes in HIV/AIDS prevalence where increased risk has shifted from large urban settings in the Northeastern and Western U.S. to the urban and rural communities in the Deep South. Additional HIV/AIDS demographic changes include a shift from being a mainly White male disease to a one largely affecting both Black males and females [1].

Although the influential role of religion in Black culture is well represented in the literature, its interconnection with migration and sexual health, particularly among those with HIV/AIDS has not been well documented. The goal of this article is to examine the influence of recent migration patterns and religion among Blacks, as well as utilizing this relationship to develop a framework for prevention strategies in sexual health including HIV/AIDS. Specifically, the authors of this paper will provide: 1) an overview of historical and current migration patterns of U.S. Blacks and their sociological implications; 2) an overview of the current HIV/AIDS epidemic among Blacks in the Deep South; and 3) give a description of a framework that connects migration, religion, and sexual health for building HIV/AIDS prevention capacity in Blacks in the Deep South using case studies to support the framework. It is hoped that the paper will not only provide a platform for strategies designed to mitigate HIV/AIDS health disparities, but also provide a model for building prevention capacity for other health disparities in the Deep South.

## 2. Overview of Black Migration and its Sociological Implications

After slavery through the signing of the Emancipation Proclamation and Post-Reconstruction period of American history, there was evidence that more than 90% of Blacks lived in the South [2]. Yet the decline in European immigration combined with industry growth at the start of World War I ushered a shift in the U.S. regional concentration of Blacks living in America. Additionally, the plausible fact that a large number of Blacks in the Deep South were still burdened with oppressive Jim Crow legislation and the exploitative economic limitations of sharecropping also contributed to the demographic shift in the early 20^th^ century. Moreover, the 1920s drop in cotton's price further hampered the economic viability of being a sharecropper in the Southern U.S. agrarian economy, which increased the attractiveness of Midwestern and Northern U.S. industries looking to hire new employees. These previously discussed pattern of events and policies led to significant migration movements of Blacks from the South to other U.S. regions [3]–[5].

In his book, *The Promised Land*, Nicholas Lemann wrote “in all, between 1910 and 1970, about 6.5 million Blacks migrated to the northern U.S. The Black Migration was one of the largest and most rapid mass internal movements of people in history-perhaps the greatest not caused by the immediate threat of execution or starvation. In sheer numbers it outranks the migration of any other ethnic group-Italians or Irish or Jews or Poles-to this country” [3].

Since the 1970s, the demographic shift initiated at the start of World War I has reversed, with large groups of Blacks moving from large urban Midwestern and Northern cities like New York, Chicago and Detroit back to areas of the U.S. Deep South [6],[7]. These more recent reverse migration trends increased the population of Blacks in the South, by roughly 1.7 million between 2000 and 2010. About 75% of recent southern U.S. population density growth among Blacks occurred in metropolitan areas such as Atlanta, Dallas, Houston, Miami, and Charlotte, North Carolina. About 57% of Blacks in America now live in the South, which appears to be one of the largest and more remarkable demographic shifts since 1960s [8].

The reasons for the population reversal may largely be related to factors associated with the first large Black migration. More specifically, the lower cost of living and increased job opportunities in the South, combined with the economic decline of manufacturing industries like the U.S. car industry in Detroit might make the southern U.S. region more attractive to Blacks despite its racially oppressive history [9]–[11]. Additionally, “reverse migrants” appear to be more educated [6],[12],[13], which may be associated with an increased locus of control and perception of ability to overcome any potential racially-charged cultural challenges. Though in contrast to common stereotypes associated with the Deep South culture, some may be moving for access to more culturally diverse experiences, more integrated neighborhoods and schools and more culturally unique attractions [14]. Reverse migrants may also see the migration shift as a maturation phenomenon where their search for living out their American dreams includes coming back to their ancestral homelands [15].

For some reverse migrators, the move back to the U.S. southern region may be related to the perception of the political and structured environment being “safer” now compared to the post-emancipation and civil rights era. For others, the move back to their ancestral roots may stem from the desire to be closer to family because of parental illness, land inheritance, or and the increased sense of community often attached to the Southern culture. Recent research suggests that Blacks following homeplace ties to the South may be declining in the movement, since Blacks born outside of the South now have a greater rate of migration to the South than those originally born in the South [12]. Other Blacks who are moving without family ties to their destination could be routed to their new southern home through chain migration [16] movement in which prospective migrants learn of opportunities, are provided with transportation, and have initial accommodation and employment arranged by means of social relationships with previous migrants, job transfers, ties to southern colleges, and connections to black media [17].

## 3. Brief Overview of HIV/AIDS Epidemiology Among Blacks

At the same time that reverse migration patterns among Blacks in U.S. are occurring during the late 20^th^ and early 21^st^ centuries, the demographic profile of the HIV/AIDS epidemic has shifted from a largely White gay male disease to a predominately Black male and female disease. HIV and AIDS rates are concerning illnesses in Black communities, threatening the health, well-being, and potential of Black men and women across the U. S. While prevention efforts have helped to maintain stability in the overall level of HIV infections, Blacks continue to face the most severe burden of HIV and AIDS of all racial/ethnic groups in the nation. Compared with other races and ethnicities, Blacks account for a higher proportion of HIV infections at all stages of the illness-from new infections to AIDS deaths. Blacks also accounted for an estimated 44% of all new HIV infections among adults and adolescents (aged 13 years or older) in 2010, despite representing only 12% to 14% of the U.S. population. Moreover, Black men accounted for 70% of all new infections among Blacks and have a rate of infection that is six and a half times higher than that of white men. The majority of new HIV infections are in men who have sex with men (MSM); new infections among young (ages 13–29) MSM increased by 48% from 2006-2009. For women, the racial disparity is even greater. The estimated prevalence rate of new HIV infections for Black women (38.1/100,000 population) was 20 times as high as the rate for white women, and almost five times as high as that of Latinas. HIV was also the ninth leading cause of death for all Blacks at the end of 2008 and the third leading cause of death for all Blacks aged 35–44 [18].

## 4. Overview of HIV/AIDS in the Deep South

In addition to ethnic/racial shifts, there have been geographic and population changes over the course of the HIV/AIDS epidemic. In the early stages of the HIV/AIDS epidemic (circa 1980s), the epidemic predominately affected gay White men living in large metropolitan cities form the Northeast and West regions of the U.S. [19]. Currently, the Deep South (including the states of Louisiana, Alabama, Mississippi, Georgia and South Carolina, and portions of Texas, Arkansas, Tennessee, and Florida [20]) makes up the region with the highest percentage of Blacks in the country and carries a disproportionate burden of HIV/AIDS [21]. Additionally, new HIV/AIDS cases are more likely to occur among people living in Southern U.S. non-metropolitan areas than in any other region in the country [21]. Sixty-five percent of all rural AIDS cases are in the South.

The state of Alabama reflects these ethnic/racial as well as geographic and population changes in the HIV/AIDS epidemic. In Alabama, Blacks account for 69% of new cases, yet they comprise only 26% of the state's population [22]. Alabama also has the 11^th^ highest HIV infection rate in the country and the eighth highest AIDS related death rate [23],[24]. Greene, Hale, and Lowndes counties in Alabama have recently emerged as “hot spots” for new HIV diagnoses. These three counties have very small populations between 9,045 and 15,760 citizens [25].

Factors related to understanding the dynamics of the HIV/AIDS epidemic, particularly in the rural Deep South include: lack of access to screening and healthcare, racism, lack of trust and suspicion, religious beliefs, homophobia, and poverty [26]–[29]. Existing literature suggests that culture in rural communities is more conservative than urban areas, valuing self-reliance and religiosity, along with a negative view of homosexuality, resulting in men fearing to be labeled as homosexual [30]–[32]. Religiosity has also been associated with negative attitudes toward individuals with HIV/AIDS and is a major barrier to both acceptance and adoption of prevention messages and interventions in rural areas, especially in many Black communities [27]. In addition, there appears to be ongoing cultural distrust of the medical community, particularly as it relates to HIV conspiracy theories, stemming in part from the infamous Tuskegee Syphilis Study conducted in the early 1900s [33].

## 5. The Black Church and Sexual Health

Scholars have long recognized the Black Church as one of the most important institutions found in the Black community, because of the absence of interference from Whites in the religious activities and expressions of Blacks after slavery. This social institution historically addressed and met the economic, social and political needs of the Black community [34]–[38]. These specific issues addressed by the Black Church surpassed the geographic limits of the South. For example, Williams described a Black Church in Pittsburgh, Pennsylvania welcoming migrants from the South, along with addressing the needs of its poor members and giving them purpose through social status received through religious titles [38].

In addition to its social role, there is evidence that the Black Church has influenced the sexuality and sexual health of its members. Historically, the Black Church has encouraged sexual silence for men and women alike. This silence is rooted in the history of early colonization of the United States, during a time when Black women were viewed as hypersexual. This mythology surrounding women in slavery was suggested to be a barrier to seeing their humanity [39],[40]. In addressing this hypersexual stigmatized identity, Black religious institutions encouraged women to create an invisibility and silence around their sexuality. Higginbotham notes, “The church played the single most important role in influencing normative values and distinguishing respectable from non-respectable behavior among working-class Blacks. Churches and households, both rejecting the worldly attractions of male social space, signified sacred space. Women who strolled the streets or attended dance halls and cheap theaters promiscuously blurred the boundaries of gender” [41]. It is believed that this sexual silence still has a place in the Black Church today [42],[43].The lack of discussions centered on sexuality in Black communities continually challenge women because of the limitations imposed by the Black Church.

Documented in the contemporary literature is again the encouragement of abstinence and married, heterosexual relationships. In addition, homosexuality is discouraged, as Frederick and Douglas note that homophobic messages conveyed through the Black Church are widespread, with homosexuality viewed as a sin. Pertinent to the topic of this manuscript, some Black church members view HIV as a “punishment from God” due to the fact that homosexuality perceived as immoral [40],[43]. As such, homophobia within the Black Church is also believed to be a factor contributing to sexual silence. Interestingly, the sexual silence and homophobia in the Black Church may have unintentionally led to individual interpretations of Biblical messages among its members.

For example, these interpretations, on one hand, may have increased the odds of inclusion among sexual minorities. For instance, Frederick notes in her ethnography of married and single Black women's membership at Black churches in rural North Carolina that while messages of homophobia are received and imbedded into personal beliefs, these Black women interviewed regularly interacted socially with homosexual minorities. These women who participated regularly in the Black Church had difficulty adopting homophobic attitudes that apply to gay and bisexual family and friends as ordained by the Black Church. Participants instead viewed those in their social networks as a whole person and not merely their sexual preferences [40].

On the other hand, these personal interpretations may have also led to increased high risk behaviors among Black church members. Valera and Taylor present a complex example of sexual behaviors of men who attend church and increase HIV risk of Black women. More specifically they describe nine married men associated with the Black Church who identified themselves as heterosexual, but also engaged in same-sex activity within the past six months of their interviews. Some participants served in leadership positions at the churches they attended. When asked how they reconcile their sexual behavior to include both their wives and secret male partners, participants noted that the Black Church's framework that encourages members to “Hate the Sin but not the Sinner” made it possible for men engaging in same-sex relationships to continue attending church. They believed that God would forgive them for their behavior, and noted that their spiritual relationship was personal, hence encouraging greater secrecy of their private sexual relationships [44].

In addition, Frederick highlights the increased risk related to sexual silence among Black women attending Black Churches. While single women were aware of the Biblical teachings regarding abstinence, they revealed that they used their personal experience and faith to make decisions regarding their sexuality, whether they were married or not. Furthermore, the church's teaching on abstinence appears to have minimal influence on the likelihood of these single women engaging in sexual activity [40].

While sexual silence has led to different outcomes as just illustrated, there is a need to overcome these barriers to engage in the HIV epidemic. Because of the HIV epidemic, these conversations are imperative to have in both Black Churches and local communities [39],[43].

## 6. Black Migration and Black Religiosity

Black migration has had a mixed relationship with Black churches and spirituality. The Great Migration, like other historical events affecting the Black community, was a source for Black religiosity to provide cultural, social, financial, and organizational tools to help Blacks engage in politics and community action [45]–[47]. Some Black ministers defined the Great Migration as a “divine plan” for Black people, while others told their congregants not to move north. Despite mixed responses among clergy leaders, Black ministers gained popularity during the Great Migration as its topic engaged congregants and helped labeled the Black Church as the social epicenter among Blacks [48],[49],[50]. The topic of migration was so strong that some Black ministers followed their congregants after they moved, while some congregations followed Black ministers that moved north.

Black ministers also worked to connect new migrants with resources during the Great Migration [49],[51],[52]. For instance, pastors worked closely with labor recruiters to get migrants into jobs. They connected migrants to housing in the cities. Pastors also worked with politicians to connect them to Black voters. Given their status in the community, they were a central resource for connecting the community of migrants with the broader community and vice versa.

Research on religion in the Black reverse migration is less developed. Stack discusses a migrant who moves to the South in part because of a spiritual experience in her church in Brooklyn, New York [15]. Pendergrass provides evidence that Blacks, especially Black women, draw greatly on spirituality as part of their migration journeys. Migrants consulted Black pastors in the North, Midwest, and West to provide spiritual confirmation for their journeys. In addition, migrants joined Black churches after arrival to get a sense of connection to the community, to get spiritual support, to get contacts for employment and business, and more [53]. Yet, there are limits to the reach of Black churches in the reverse migration, as reverse migrators appear more critical of Black churches. Investigation of these and other dynamics of religion in the Black reverse migration need more development. We especially know little about the complexities of religion and reverse migration in the rural, Deep South.

Religious beliefs rooted in Southern Black communities influence health beliefs and health behaviors in this community. For example, some rural Southern Blacks believe that God is the ultimate cause of all illness and misfortune, which appears to be a sign of increased external locus of control. Counterintuitive to what one may believe, individuals “who violate the moral codes of the community or who indulge in improper or “sinful” behavior” [54] may continue to do so because they may perceive that their actions are mediated by forces beyond their control. Furthermore, elderly rural Blacks living in the South use both traditional and biomedical health orientations and practices to maintain a healthy lifestyle. Traditional practices for the maintenance of health include prayer and reading the Bible [55].

## 7. The Stigma Fear Denial (SFD) Framework for HIV/AIDS Prevention in the Deep South

Because of the emerging and shifting demographics of HIV/AIDS in the U.S. over the last three decades, characterization of the epidemic in the rural Deep South is relatively new and limited. However, one of this manuscript's authors developed a theoretical framework for preventing HIV/AIDS in the rural South Blacks based on experiential HIV prevention activities [27]. The framework first identifies three components that serve as major barriers in conducting HIV prevention: stigma, fear and denial (SFD). After identifying barriers, the theoretical framework uses an empowerment model to increase HIV prevention through three major components: increasing community empowerment, cultural competence and social action. Use of this model in targeted groups could be used to build capacity of the community to conduct prevention activities. The authors suggest that use of this framework specifically in reverse migrators in both the pastoral and consumer (HIV+) groups might be useful.

### 7.1. Case Studies

In order to illustrate the interconnection between HIV prevention using the SFD Prevention framework and knowledge regarding reverse migration, the Black church, and sexual health in Black communities, we present the following two cases in the Deep South. The first case involves findings from interviews with eight Black Baptist pastors in rural Alabama about their current HIV/AIDS prevention activities within their churches and their own personal interests in HIV prevention activities. The second case highlights activities of two AIDS Service Organizations that conduct faith-based prevention activities and have moved their services to the Deep South in order to accommodate changes in the HIV/AIDS epidemic.

#### 7.1.1. Migration and HIV/AIDS Capacity Building in Rural Alabama

The first study was conducted by this manuscript's first author to assess potential barriers of HIV/AIDS prevention among Black Baptist Pastors with their congregations in rural Alabama. All Pastors were members of the New Era Progressive Baptist Conference, which has 110 member congregations. A total of eight Black pastors were interviewed in a one-to-two hour semi-structured interview. In-depth interviews were audiotaped and transcribed in order to conduct constant comparative analysis. Open coding was used to identify key concepts and categories for summarization and analysis of data. All interviews were conducted by the research team in the offices or homes of the participants. Informed consent was obtained by all participants before the start of the interview. Demographic information of each Pastor was also collected.

All eight of the Pastors interviewed were Black, male, and married. Seventy-five percent of the participants (6/8) were over the age of 50. One of the participants was a college graduate with some graduate school and half (4/8) attended some college. All men served as pastors of churches considered rural as designated by the U.S. rural-urban commuting area codes [56]. Several of the participants had received HIV testing with most receiving testing because of a secondary job mandate for health insurance screening. A vast majority of participants (88%) considered themselves to be in full-time ministry with half of them in various occupations including funeral director, mechanic, management and the auto industry. Of those interviewed, only two had conducted at least one HIV prevention activity in their congregation in the last five years. Despite this, all of the men (8/8) stated interest in conducting HIV prevention activities in their churches in the future.

**Figure 1.**
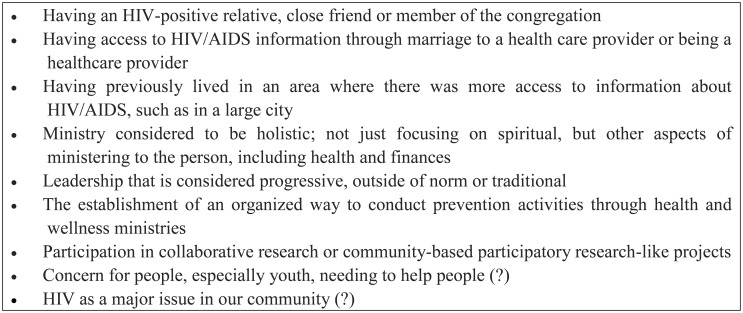
Positive Influencers of HIV Prevention in Pastors

Initially, the proposed study sought to explore potential barriers for rural Black pastors to conduct HIV prevention activities in their congregations and communities including disinterest in presenting this information to church members. However as the study unfolded, the researchers realized that because most of the study participants were indeed either participating or very interested in participating in HIV prevention activities, we needed to expand beyond looking at barriers to HIV prevention activities to begin to look at what positive influencers might encourage a Pastor to become involved in HIV/AIDS prevention. Figure 1 summarizes some of the positive influencers (hypothesized) that we reported as a result of the study.

One of the most interesting positive influencers that we captured from this study was that several of the participants were reverse migrators. Although all participants were born and raised in Alabama, several had moved away to other geographical areas of the country, often larger metropolitan areas, and eventually moved back home. Reports of reverse migration to suburban and rural Deep South have not been extensively reported in the literature, especially as it relates to HIV/AIDS prevention (as an exception see reference [15]). Additionally, we theorized from our study that our small pool of Pastors, most of whom were reverse migrants, may have more propensity than native Southerners to be open to conducting HIV prevention within their congregations and their local community. Therefore, we also suggest that this reverse migration group could potentially be utilized to increase HIV/AIDS prevention capacity in Southern rural Black communities where they now live.

#### 7.1.2. Migration and Faith-based Capacity Building in Virginia

It is believed that Northern and Western U.S. faith-based communities replied to the epidemic more readily because of its initial exposure to HIV in the early 1980s. However, because of the changing epidemic, programs are now being incorporated in faith-based institutions in the South. Fortunately, there is one woman, Pernessa C. Seele, well known for introducing HIV prevention into local churches and communities. She migrated from the North to the Southeastern United States to work on promoting conversations surrounding sexuality and HIV in the Black Church.

The Balm in Gilead, a non-profit organization that addressed the HIV/AIDS epidemic early on in New York, overcame the sexual silence of the Black Church in an effort to address the friends and families of HIV positive community and church members [57]. The founder, Pernessa C. Seele, was able to overcome the barriers typically found in the Black Church through a reframing of AIDS from a deviant to a more acceptable disease for those attending the church. Participating Black Churches adopted an emphasis of prayer for those infected. Steele initiated this by organizing the Harlem Week of Prayer. Also, an emphasis to care for the ill and suffering was adopted. Finally, provision of HIV/AIDS educational material was made available to participating Black Churches.

The culturally competent approach that enabled the Black Church to incorporate Biblical mandates of prayer and caring for the sick enabled congregants to adopt a more positive view of those infected with HIV/AIDS. In addition, the community concern of educating members allowed for church members to desire greater access to HIV/AIDS educational materials. According to the Center for Disease Control and Prevention, the Balm of Gilead serves more than 2.5 million faith community members [58]. Since the organization's inception, their reach has moved from New York to all across the country. Additionally, Seele has moved the Balm of Gilead headquarters to Richmond, Virginia in order to better address the HIV epidemic now found in the Southeastern United States, including the rural South [59].

## 8. Discussion

We theorize that the combination of reverse migrators and faith-based approaches to HIV prevention could be used to build capacity for prevention, particularly in the rural Deep South where both have tremendous influence and impact in the South. This review paper takes a critical look at all these interrelationships through the conceptual application of an empowerment HIV prevention framework supported by case studies. The first case study implies that reverse migrators in either faith based settings or people living with HIV/AIDS (PLWHA) may be more willing to engage in HIV prevention than non-migrators who live in the Deep South. The reasons for this are unknown. The authors theorize that reverse migrators may have the advantage of greater access to HIV/AIDS information, AIDS service organizations, AIDS advocacy, and HIV/AIDS treatment and prevention activities which might make them more prepared to engage in HIV/AIDS prevention activities. Additionally, there may be increased access of reverse migrators with persons who are openly living with the disease. For example, many of the pastors were influenced to get involved because they personally knew someone affected by the disease.

Ironically, the linkage of reverse migration and HIV/AIDS in this group of pastors also seemed to correspond to data from other studies by this manuscript's first author where “reverse migrators living with HIV/AIDS (RMLWHA)” often came back to the Deep South because of their desire to be close to family. For example, focus groups conducted with older infected persons over the age of 50 revealed that several of the participants were “reverse migrators” who often moved back home to the rural South from larger cities like Atlanta, New York, Washington D.C. and Los Angeles in order to be closer to family for support [60].

We also noticed that often these RMLWHA were often more knowledgeable about their disease than non-migrators, which empowered them to maximize management of their disease as copartners with their health care providers and act as peer leaders for others infected. Additionally, they appeared to be more open about their diagnosis in order to disclose to a larger network of friends, family, and the larger community. Many were active in support groups, and peer network groups, and community-based HIV/AIDS prevention activities where they often took leadership roles. Additional studies for reverse migrators in both pastors and PLWHA are needed to test these theories within the framework.

The second case study illustrates the shifting of faith based organizational capacity around HIV prevention from places like New York City where there was an initial need for addressing the HIV epidemic to places in the South like Virginia, where the need for HIV capacity building is emerging. In many ways, these faith-based organizations are “reverse migrators” in shifting initial needs around the epidemic, to move to where the need is now greatest. The use of both groups of reverse migrators as well as shifting or migrating organizational capacity where it is needed could be powerful in building the capacity of African American communities to prevent HIV/AIDS, particularly in the rural Deep South.

This paper presents a theoretical framework and data, which loosely correlates the timing of the increasing HIV epidemic in the rural South, reverse migration and the importance of faith-based approaches in HIV prevention. This review paper appears to be one of the first of its kind to make these correlations. Because the relationships are not directly measured, preliminary and qualitative in nature, this is a limitation. Despite this limitation, we believe that because HIV/AIDS continues to increase in rural Blacks in the Deep South, studies which can further investigate the interrelationships between all of these factors in building capacity to address the epidemic in Blacks in the rural Deep South needs to be further explored.
